# Genome-wide identification, expression analysis of WRKY transcription factors in *Citrus ichangensis* and functional validation of *CiWRKY31* in response to cold stress

**DOI:** 10.1186/s12870-024-05320-0

**Published:** 2024-06-28

**Authors:** Jing Qu, Peng Xiao, Ze-Qi Zhao, Yi-Lei Wang, Yi-Ke Zeng, Xi Zeng, Ji-Hong Liu

**Affiliations:** National Key Laboratory for Germplasm Innovation & Utilization of Horticultural Crops, College of Horticulture and Forestry Sciences, Huazhong Agricultural University, Wuhan, 430070 China

**Keywords:** Ichang papeda, *Citrus ichangensis*, WRKY, Cold stress, Genome-wide analysis

## Abstract

**Background:**

Ichang papeda (*Citrus ichangensis*), a wild perennial plant of the Rutaceae family, is a cold-hardy plant. WRKY transcription factors are crucial regulators of plant growth and development as well as abiotic stress responses. However, the *WRKY* genes in *C. ichangensis* (*CiWRKY*) and their expression patterns under cold stress have not been thoroughly investigated, hindering our understanding of their role in cold tolerance.

**Results:**

In this study, a total of 52 *CiWRKY* genes identified in the genome of *C. ichangensis* were classified into three main groups and five subgroups based on phylogenetic analysis. Comprehensive analyses of motif features, conserved domains, and gene structures were performed. Segmental duplication plays a significant role in the *CiWRKY* gene family expansion. *Cis*-acting element analysis revealed the presence of various stress-responsive elements in the promoters of the majority of *CiWRKYs*. Gene ontology (GO) analysis and protein-protein interaction predictions indicate that the CiWRKYs exhibit crucial roles in regulation of both development and stress response. Expression profiling analysis demonstrates that 14 *CiWRKYs* were substantially induced under cold stress. Virus-induced gene silencing (VIGS) assay confirmed that *CiWRKY31*, one of the cold-induced WRKYs, functions positively in regulation of cold tolerance.

**Conclusion:**

Sequence and protein properties of *CiWRKYs* were systematically analyzed. Among the 52 *CiWRKY* genes 14 members exhibited cold-responsive expression patterns, and *CiWRKY31* was verified to be a positive regulator of cold tolerance. These findings pave way for future investigations to understand the molecular functions of *CiWRKYs* in cold tolerance and contribute to unravelling WRKYs that may be used for engineering cold tolerance in citrus.

**Supplementary Information:**

The online version contains supplementary material available at 10.1186/s12870-024-05320-0.

## Background

Citrus is an important source of human nutrition with high economic value; however, different biotic and abiotic stresses threaten production and limit its distribution [1]. Ichang papeda (*Citrus ichangensis*), a wild plant of the Rutaceae family, originates from the southwestern and middle-west regions of China and has a sporadic distribution at high elevations or humid conditions in mountainous regions [2]. It exhibits superior tolerance to extreme frosts and damp environmental conditions [3], making it a valuable germplasm for investigating stress response mechanisms. In this regard, identification of cold-responsive genes of *C. ichangensis* is of great importance to improve the stress resilience of citrus.

Fluctuations in the biotic and abiotic environment are detected and transmitted through various signaling pathways, ultimately leading to alterations in gene expression [4]. Being immobile plants cannot evade unfavorable circumstances by relocating to more suitable locations. They have to rely on particular physiological or developmental modifications to react and adapt to harsh environments [5]. The WRKY transcription factors, one of the most extensive families of regulatory proteins in plants, play crucial roles in governing the plant response to stressful conditions [6, 7]. The WRKY proteins are defined by the presence of highly conserved seven amino acid sequence (WRKYGQK), known as the WRKY domain surrounded by a C2H2 or C2HC zinc finger motif [8]. Both the peptide sequence and zinc finger motifs are essential for the sequence-specific recognition and high affinity of the WRKYs to their canonical *cis*-acting element W-box sequence (T)TGAC(C/T) [9].

Based on the zinc-finger motif structure and the number of WRKY domains, WRKY proteins are classified into three major groups [10]. Group I include proteins containing two WRKY domains with a C2H2 motif, whereas Group II members possess a single WRKY domain along with a C2H2 motif. In addition, the Group II proteins can be further categorized into five subgroups, IIa, IIb, IIc, IId, and IIe. Group III WRKY proteins possess a single WRKY domain and a C2CH motif.

As one of the largest families of TFs with diverse regulatory functions, the *WRKY* genes are closely associated with the regulation of stress responses in higher plants [11]. It has been reported that most plant WRKY proteins function as positive regulators in environmental adaptation. For example, VqWRKY31 of grapevine directly regulates *STS9* and *STS48*, two genes involved in stilbene synthesis, to regulate powdery mildew resistance [12]. CdWRKY2 of bermudagrass has been demonstrated to play a role in regulation of sucrose biosynthesis, in conjunction with the CBF signaling pathway, to increase cold tolerance [13]. In addition, some plant WRKY proteins have been reported to function as negative regulators of stress defense. For instance, OsWRKY63 of rice negatively regulated chilling tolerance through the OsWRKY63-OsWRKY76-*OsDREB1B* signaling cascade [14]. In another study, overexpression of *OsWRKY10* resulted in the accumulation of reactive oxygen species (ROS) in both rice chloroplasts and apoplasts [15]. Moreover, *OsWRKY5* acts as a repressor against ABA-triggered drought resilience [16]. Additionally, ZmWRKY20 and ZmWRKY115 reduced the capacity of maize to tolerate high salinity by repressing *ZmbZIP111* expression [17].

Since WRKY family genes play a significant role in plant stress resistance [18], structural characteristic and functions of WRKY transcription factors from various species have been identified so far. For example, 74 *WRKY* genes have been identified in Arabidopsis [19], 103 in rice [20], 188 in soybean [21], and 58 in maize [22]. Genome-wide characterization of WRKYs has also been conducted in horticultural plants, which revealed presence of 81 *WRKY* genes in tomato [23], 61 in cucumber [24], 127 in apple [25], 103 in pear [26], and 54 in pineapple [27]. However, there is limited information on the number, characteristics, evolutionary connections, and expressions of *WRKY* genes in *C. ichangensis*, which to some extent impedes our understanding on the role of WRKY in regulation of cold tolerance in Ichang papeda.

In this study, 52 *WRKY* genes were identified in *C. ichangensis* (names as *CiWRKYs*), which were categorized into three main groups and five subfamilies. A comprehensive analysis was conducted to characterize the exon-intron, motif composition, subcellular localization, gene duplication, chromosome distribution, and phylogenetic and synteny analyses. Gene Ontology (GO) annotations and promoter analysis were also carried out to better understand the biological functions of *CiWRKYs.* Moreover, the expression patterns of *CiWRKYs* under cold treatment were assessed using RT-qPCR, and the transcript abundance of 14 most cold-responsive genes was examined based on the RNA-seq data of *C. ichangensis*. Finally, the role of *CiWRKY31* in cold tolerance was experimentally verified by using VIGS (virus-induced gene silencing). Taken together, this study uncovers the functional roles of *WRKY* genes in *C. ichangensis* under cold stress and provides valuable insights for exploring specific *CiWRKY* genes to enhance cold tolerance in *Citrus*.

## Materials and methods

### Plant materials and cold treatment

*C. ichangensis* seeds harvested from the Citrus Breeding Centre at Huazhong Agriculture University were sown in soil pots and grown for three months at 25 °C. For cold treatment, 3-month-old plants were placed in a low-temperature incubator set at 4 °C. The leaves were collected at specific time points (0 h, 6 h, 24 h, 72 h, and 120 h), immediately frozen in liquid nitrogen and stored at − 80 °C for further analysis.

### Sequence retrieval

The *Citrus ichangensis* genome sequence was downloaded from the Citrus Pan-genome to Breeding Database (http://citrus.hzau.edu.cn/) [28]. The WRKY domain (PF03106) hidden Markov model file was acquired from the Pfam protein family database (http://pfam.xfam.org/) [29]. The putative *CiWRKY* genes were identified using the HMMER3.3.2 program (https://www.ebi.ac.uk/Tools/hmmer/search/hmmscan) [30]. Based on the filtered results from the HMMER database, all identified genes were then investigated utilizing two online software tools. SMART (http://smart.embl.de/) [31] was used to verify that the candidate CiWRKY proteins containing the conserved WRKY domains. The ExPASy website was utilized to evaluate the amino acid length, molecular weight, and isoelectric point of the protein (https://web.expasy.org/protparam/). The PSORT software was employed for the prediction of subcellular localization (https://www.genscript.com/psort.html).

### Sequence analysis

The WRKY domains of the identified *C. ichangensis* WRKY proteins were aligned with Clustal default parameters to generate protein sequence alignments. The inferred amino acid sequences within the WRKY domains were subsequently manually refined with the GeneDoc software. The exon-intron arrangement of *CiWRKY* genes was obtained by comparing the predicted coding sequences (CDS) with their corresponding full-length counterparts using the online software Gene Structure Display Server (GSDS) (http://gsds.cbi.pku.edu.cn). The conserved motifs of CiWRKY proteins were examined using the MEME online software (https://meme-suite.org/meme/). The gene structure and conserved motif were illustrated utilizing the TBtools software [32].

### Phylogenetic analysis

The peptide sequences of the AtWRKY proteins were obtained from The Arabidopsis Information Resource (TAIR, https://www.arabidopsis.org/). The Clustal software was used to align all conserved WRKY domains. Phylogenetic tree was constructed using the neighbor-joining (NJ) method in MEGA 7.0, with 1,000 bootstrap replications [33], and a high-quality image of the phylogenetic tree was generated by iTOL (https://itol.embl.de/).

### Chromosomal distribution and gene duplication analysis

The physical location of *CiWRKY* genes chromosome was obtained using the Rag Tag software (Unpublished data). Gene duplication was assessed by MCScanX with default parameters using *WRKY* gene annotations and the whole-genome sequences of *C. ichangensis* and *Arabidopsis thaliana*, rice (*Oryza Sativa*), apple, and grape [34]. Tbtools software was utilized in constructing the circos plot.

### *Cis*-acting element analysis in *CiWRKYs* promoters

The promoter sequences 2000 bp upstream of *CiWRKY* genes were obtained by utilizing the “Sequence Fetch” tool from the Citrus Pan-genome to Breeding Database. Plant CARE (https://bioinformatics.psb.ugent.be/webtools/plantcare/html/) was utilized to analyze the ten most representative *cis*-acting elements involved in stress response of the *CiWRKY* promoters. Visualization was performed by the TBtools software.

### Protein interaction analysis and GO annotations

The program STRING 12.0 (https://cn.string-db.org/) was utilized for the prediction of protein-protein interactions, with a confidence score of > 0.4. Gene ontology (GO) annotation was performed by the phyper function in the R package and GO terms with a Q-value ≤ 0.05 were selected as overrepresented. The bubble diagram was generated using the Dr. Tom Multi-omics Data Mining System (https://biosys.bgi.com).

### RNA extraction and qRT-PCR analysis

RNA was extracted from the harvested samples using a commercially available RNA extraction kit (RN33; Aidlab Biotech Co. Ltd, Beijing, China). The extracted RNA was transformed into complementary DNA (cDNA) using HiScript III RT SuperMix for qPCR (+ gDNA wiper) (Vazyme, Nanjing, China). RT-qPCR was performed on an ABI7500 system (Applied Biosystems, Foster City, CA, USA) using AceQ SYBR Green Master Mix (Vazyme, Nanjing, China). The thermocycling conditions were 95 °C for 5 min, followed by 40 cycles of 95 °C for 10 min, 58 °C for 30 s, and 95 °C for 30 s. The 10 µl reaction mixture consisted of 5 µl of 2×SYBR Green PCR Master Mix, 0.2 µl of 10 mM primers, and 200 ng of cDNA. Three replicates were performed for each sample. *ACTIN* was used as an internal reference gene, and the relative expression level was calculated using the 2-^▵▵CT^ method [35]. The primer sequences used in this study are listed in Table S8. Expression heatmap visualization was performed by the TBtools software. Transcript profile of cold-responsive *CiWRKY* genes was carried out from an earlier transcriptome dataset [36].

### Virus-Induced Gene Silencing (VIGS)

A 300-bp partial segment of *CiWRKY31* was amplified using primers containing *BamH*I and *Sma*I restriction sites, then ligated to the pTRV2 vector (tobacco rattle virus 2) to create the pTRV2-*CiWRKY31* construct. The plasmid was introduced into the *Agrobacterium tumefaciens* strain *GV3101* by heat shock. The fusion construct (pTRV2-*CiWRKY31*) and control vectors (pTRV1 or pTRV2) were separately mixed (construction: control; 1:1) and then infiltrated into 1-month-old Ichang papeda seedlings [37]. After 3 d of growth in darkness at 25 °C, the infiltrated plants were transferred to soil and grew for 1 month. Genomic PCR was conducted to detect positive transgenic lines, while RT-qPCR was utilized to assess the transcript levels of *CiWRKY31* in the VIGS plant.

### Cold tolerance assay of VIGS plants and physiological measurements

The VIGS and control plants were treated at − 4 °C for 8 h and leaves were collected for further analysis of physiological indexes. Electrolyte leakage was measured following the method described in a previous study [38]. Chlorophyll fluorescence was assessed utilizing an IMAGING-PAM chlorophyll fluorometer (Walz, Germany), and the *Fv*/*Fm* ratio was calculated with the Imaging Win Gege software.

### Statistical analysis

The experiments were performed using a completely randomized design. Statistical differences were analyzed using the Statistical Package for the Social Sciences (SPSS) software with a one-way Analysis of variance (ANOVA) method based on the LSD test, taking *P* < 0.05 (*), *P* < 0.01 (**), and *P* < 0.001(***) as statistically significant.

## Results

### Identification of WRKYs in *C. ichangensis*

A total of 52 putative *CiWRKY* genes were obtained by HMM search against the *Citrus ichangensis* genome. SMART software tools were then used to ensure the accuracy and completeness of the HMMER search results. The 52 gene models were handpicked and designated as *CiWRKY* genes owing to the verification of fully intact WRKY domains. The *CiWRKY*s were then annotated and renamed (Table S1) through comparative analyses by blasting against the peptide sequences of Arabidopsis *WRKY* genes [18]. Their CDS ranged from 240 bp to 2736 bp, with corresponding polypeptide between 79 and 911 amino acids. The theoretical isoelectric points of the CiWRKY proteins varied between 5.01 and 9.83, and their molecular weights ranged from 8.87 kDa to 10.2 kDa. Furthermore, subcellular localization prediction suggested that all the CiWRKY proteins are located in the nucleus, in line with their typical function as transcription factors.

### Multiple sequence alignment, classification and phylogenetic analysis of *CiWRKYs*

To gain insight into the properties of the CiWRKYs, multiple sequence alignments were conducted to assess the structural features of CiWRKY proteins. A fragment of 60 amino acids containing the WRKY domains in each protein was selected for analysis (Fig. S1). Specifically, a total of 50 members were found to possess the WRKYGK domains, indicating high degree of sequence conservation among the CiWRKYs. However, it is important to note that *CiWRKY50* showed a slight variation in a single amino acid. In addition, the protein sequence of *CiWRKY52* was shorter and did not exhibit a precise alignment with that of other CiWRKY members.

**Fig. 1:**
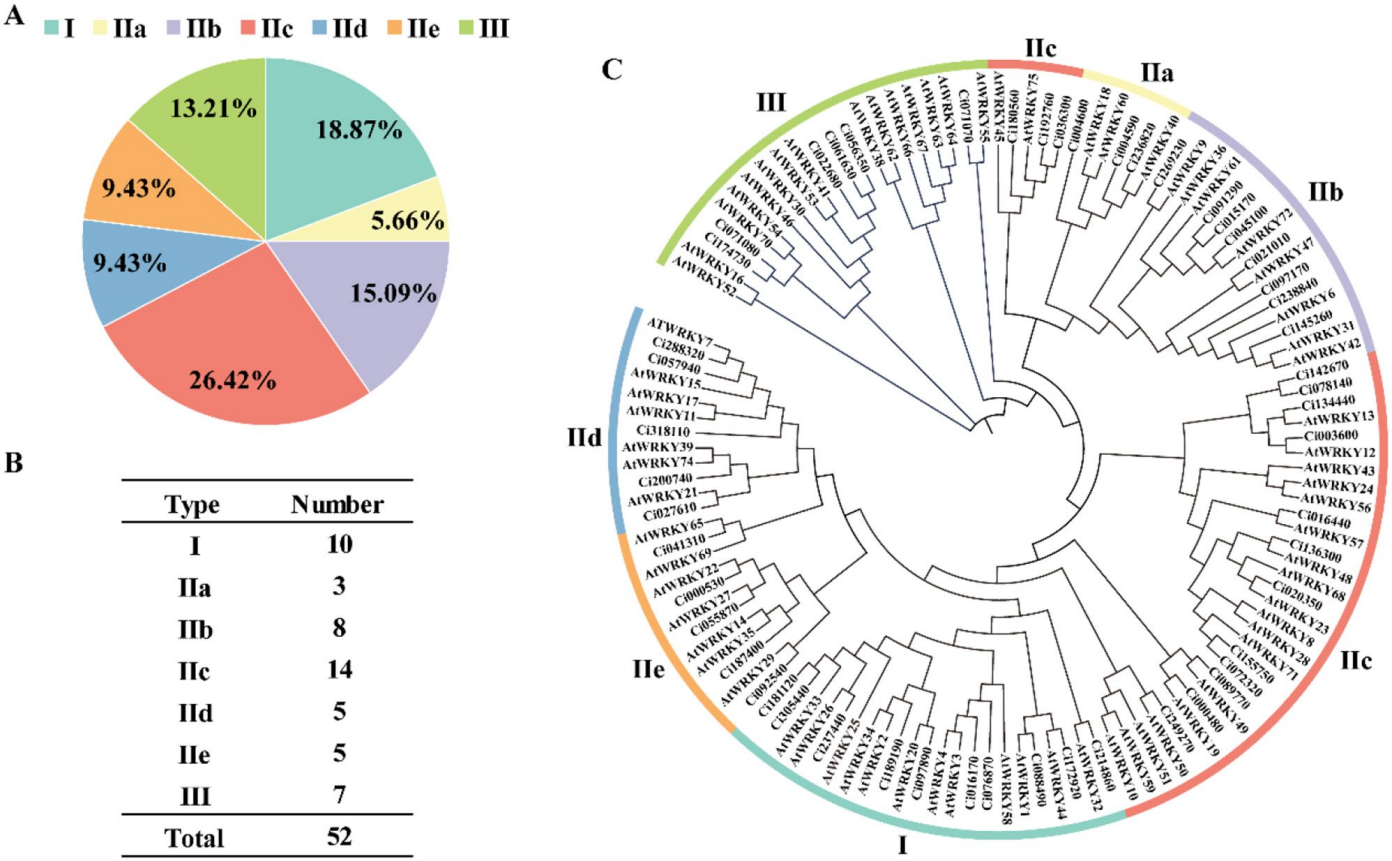
Classification and phylogenetic analysis of *CiWRKY* genes. **(A-B)** Proportion **(A)** and number **(B)** of *Citrus ichangensis WRKY* genes in each group or subgroup. **(C)** The phylogenetic tree between CiWRKYs and AtWRKYs, created using MEGA by the Neighbor-Joining (NJ) method with 1,000 bootstrap replicates. All members were clustered into Groups I, Group II (IIa, IIb, IIc, IId, IIe), and Group III

Then, based on the category of *Arabidopsis* WRKY proteins, all CiWRKY members were classified into three groups (Group I, II and III) (Fig. 1A). Ten proteins possessing two WRKY domains was categorized into Group I, while seven CiWRKY proteins were classified in Group III. Group II comprised 35 *Ci*WRKY proteins and was further classified into five subgroups, with 3, 8, 14, 5, and 5 members in subgroups IIa-IIe, respectively (Fig. 1B). Next, a phylogenetic tree was constructed using the protein sequences of CiWRKYs and AtWRKYs (Fig. 1C), which better illustrates the evolutionary relationship among the WRKY members in the two species.

### Gene structure and conserved motif analysis of *CiWRKYs*

**Fig. 2:**
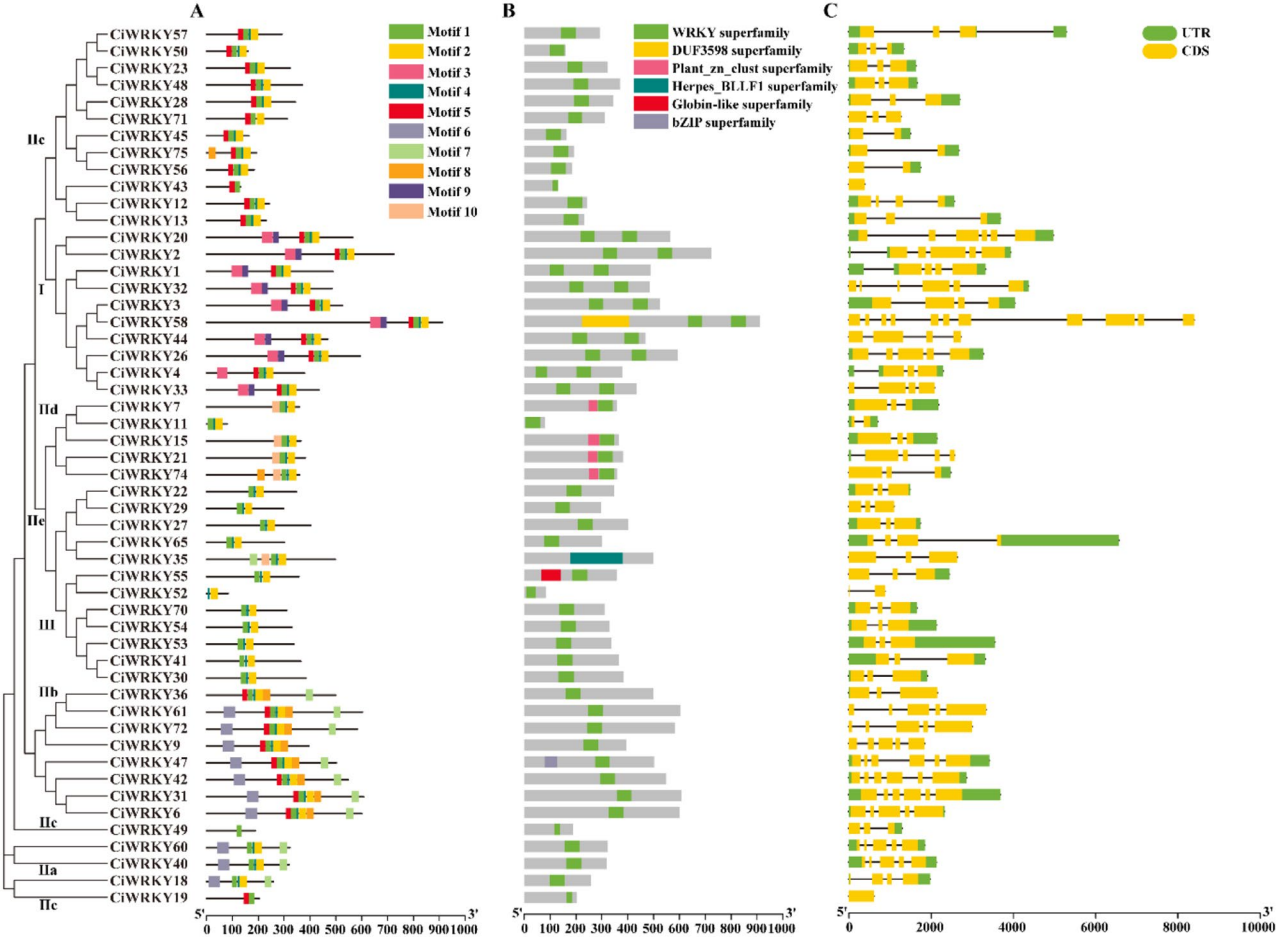
Gene structure and conserved motif analysis of CiWRKYs. **(A)** Motif composition of the CiWRKY proteins. The 10 types of motifs are displayed in boxes with different colors. The details of motif sequence were illustrated in supplemental Table S2. **(B)** Conserved domains in the CiWRKYs. The six conserved domains are shown by boxes with different colors. **(C)** Gene structure of *CiWRKYs.* Green and yellow boxes indicate the UTR and CDS regions, respectively; the black lines indicate introns. Sizes of the genes can be estimated by scale at the bottom

Gene structural diversity can provide insight into the evolution of multigene families [39]. In order to gain a more comprehensive understanding of the structural characteristics of *CiWRKY*s, we conducted analyses of conserved motifs, conserved domains and exon-intron structures to provide a comprehensive overview. A total of 10 conserved motifs were identified in the *C. ichangensis* genome using the MEME tool (Table S2). As is shown in Fig. 2A, motifs 1, 2, and 4 were extensively distributed in CiWRKY proteins. Motifs 3 and 9 were detected exclusively in the Group I members, whereas motifs 6, 7, 8, and 10 were only present in the Group II members. Of note, motif 10 was uniquely detected in subgroup IId.

Conserved domain analysis demonstrated that among the 52 CiWRKY proteins, 42 (80.8%) possessed one WRKY domain while the remaining 10 (19.2%) had two WRKY domains. Except the conserved WRKY domains, five other distinct domains were also identified in the CiWRKYs, including the Globin-like subfamily and bZIP subfamily domains (Fig. 2B).

Furthermore, we examined the exon-intron structure of each *CiWRKY* gene to obtain a more comprehensive understanding of the evolution of *C. ichangensis* WRKY family. As shown in Fig. 2C, all *CiWRKY* genes contain one to eleven exons, in which one exon was observed in two genes, two exons in five genes, three exons in 23 genes, four and five exons in eight genes, six exons in five genes, and 11 exons in one gene. Genes belonging to the same group typically exhibit the similar exon-intron structure. For instance, all members of Group III contain three exons and two introns. Overall, the arrangement of exons and intron phases is consistent with the alignment clustering of *CiWRKY* genes.

### Chromosomal distribution and synteny analysis of *CiWRKYs*

**Fig. 3:**
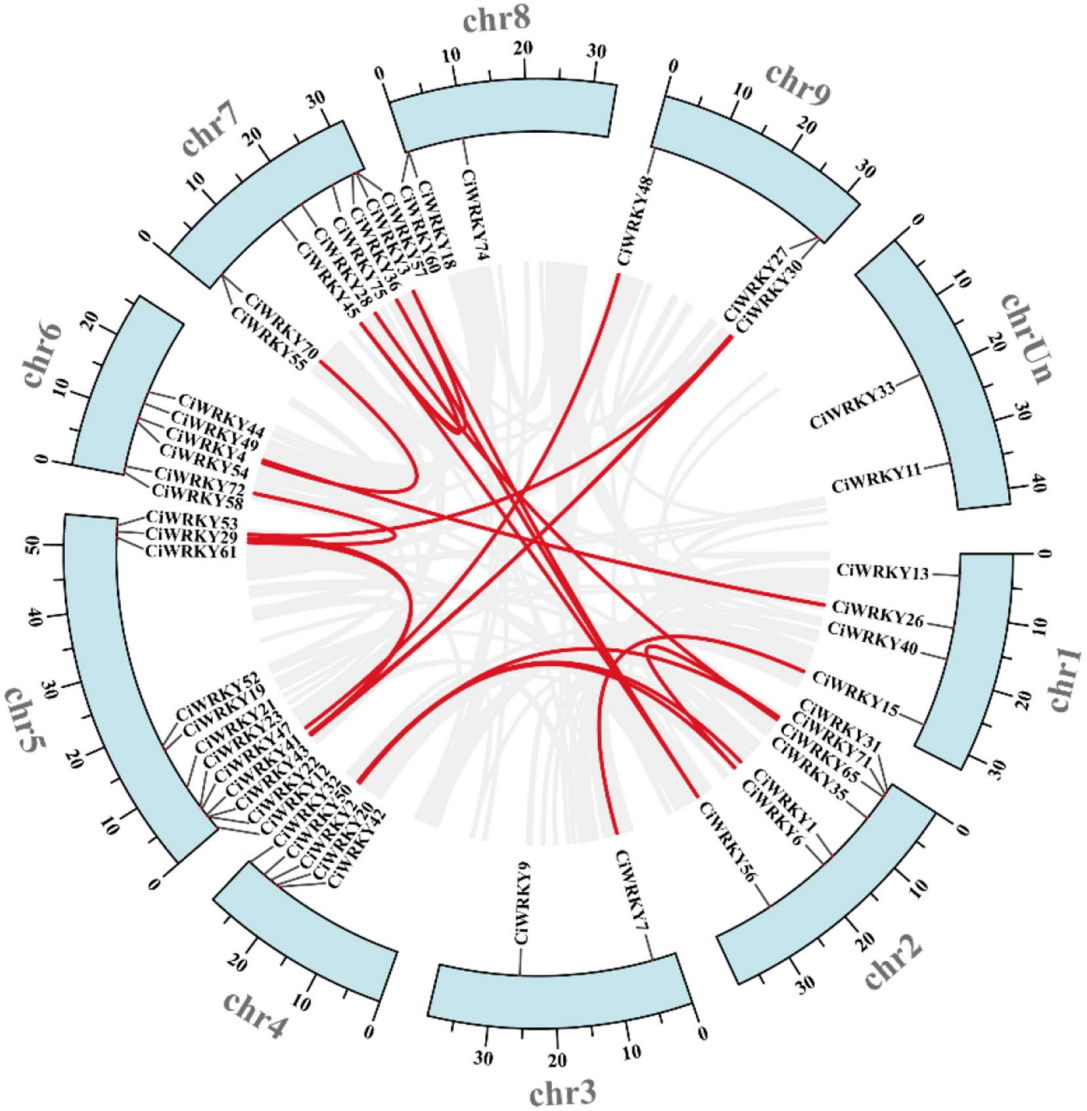
Chromosomal distribution and synteny analysis of *CiWRKYs*. The circle diagram illustrated the chromosomal location of *CiWRKY* genes and their syntenic relationships. Gray lines represented synteny blocks within the *C. ichangensis* genome, and the red lines represented *CiWRKY* gene pairs. The exact position of these *WRKY* genes was presented in supplemental Table S3

**Fig. 4:**
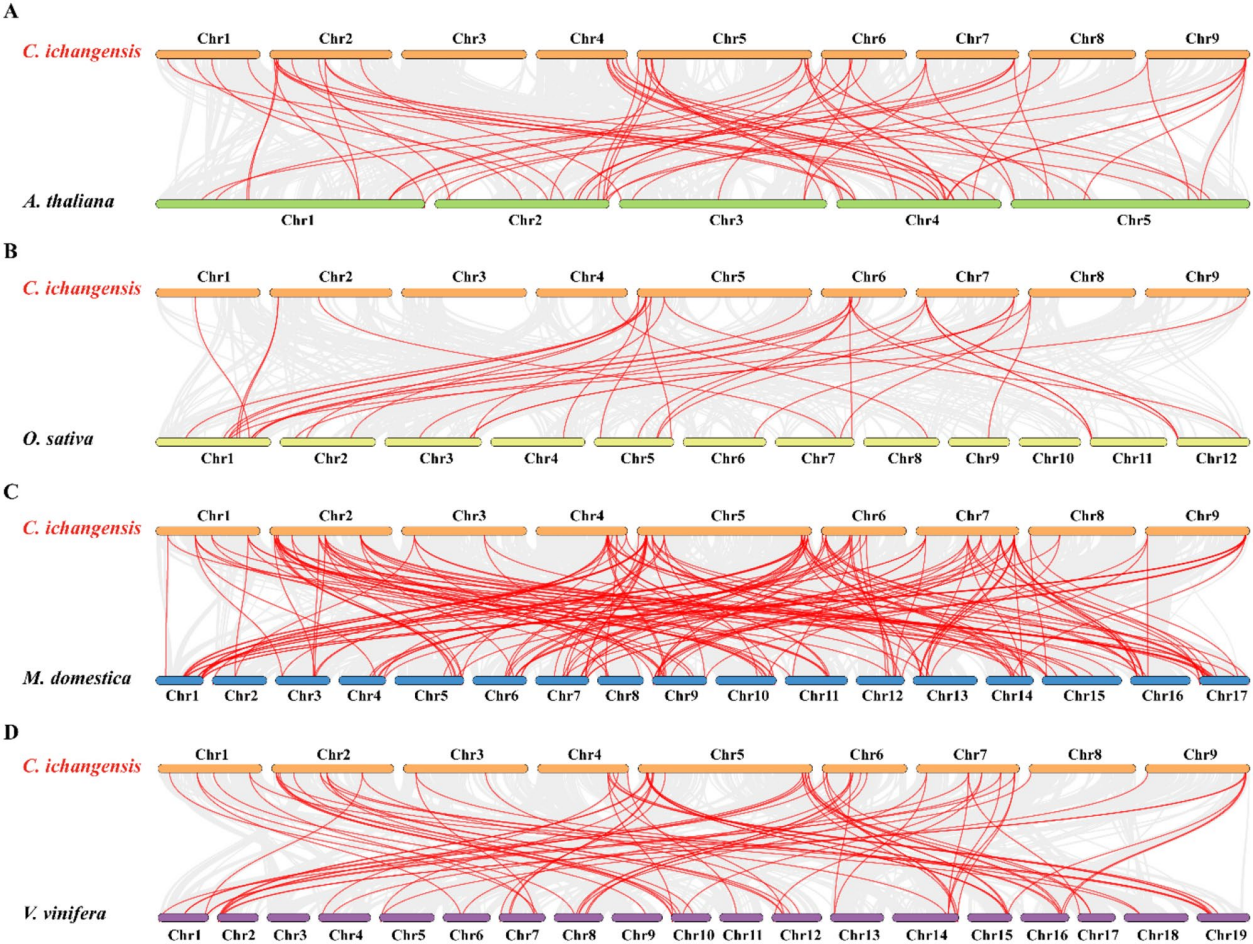
Synteny analysis of *WRKY* genes between *C. ichangensis* and other plants. **(A**–**D)** Synteny between *C. ichangensis* and *A. thaliana ***(A)**, rice **(B)**, apple (**C**) and grape **(D)**. The collinear blocks were shown with gray lines in the background, while the syntenic WRKY gene pairs were highlighted in red lines. The horizontal columns depict chromosomes with their corresponding chromosome numbers shown beside the diagram. The exact position of these *WRKY* genes was presented in supplemental Table S11–14

To identify potential gene duplication events and the phylogenetic relationships among the *CiWRKY* genes, the syntenic blocks within the *C. ichangensis* genome were examined (Fig. 3; Table S3). A total of 22 *CiWRKY* gene pairs were identified and situated on distinct chromosomes, suggesting that segmental duplications occurred in these regions, which may contribute to the *CiWRKY* family expansion. Distribution of the *CiWRKY* genes was not equal among the nine chromosomes, with the largest number (12) in chromosome 5 and the least (2) in chromosome 3. Four comparative syntenic maps were established between *C. ichangensis* and *A. thaliana*, rise, apple and grape. A total of 38 *CiWRKY* genes showed a syntenic relationship with counterparts in *A. thaliana* (Fig. 4A), 19 with rice (Fig. 4B), 44 with apple (Fig. 4C) and 43 with grape (Fig. 4D) (S11–14). The orthologous pairs between *C. ichangensis* and *A. thaliana*, rice, apple, and grape were 64, 37, 168, and 78, respectively. Certain *CiWRKY* genes were found to be orthologous with at least two syntenic gene pairs (particularly with apple *WRKY* genes), such as *CiWRKY75* (*Ci036300*) and *CiWRKY57* (*Ci016440*), suggesting that these genes may play an important role during the evolutionary process.

### *Cis*-acting elements analysis in the promoters of *CiWRKYs*

**Fig. 5:**
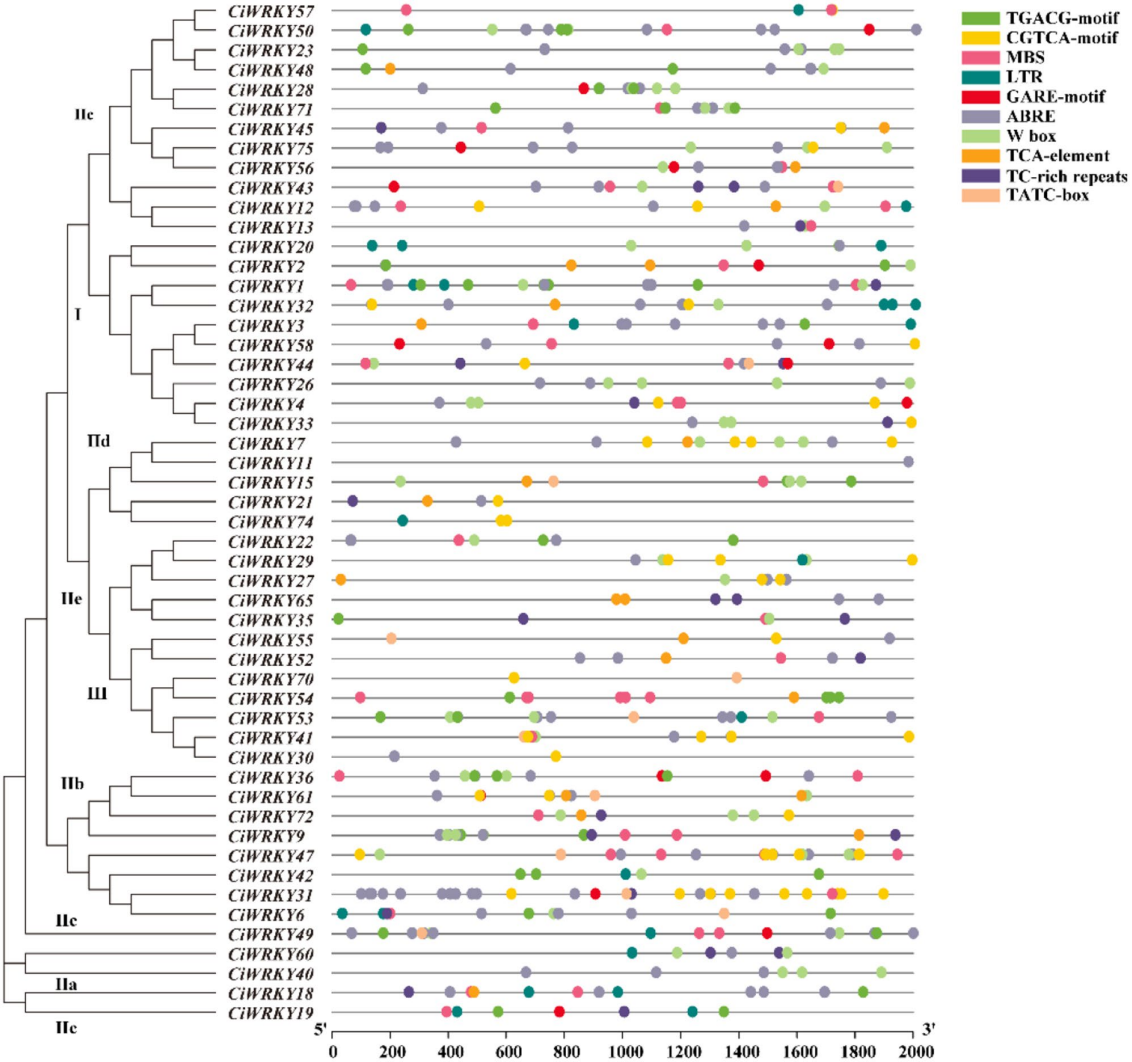
*Cis*-acting elements in the promoters of *CiWRKYs*. Ten *cis*-acting element motifs within the 2 kb of *CiWRKYs* promoter regions are highlighted and represented by boxes with different colors. ABRE: Abscisic acid response element; CGTCA-motif/ TGACG-motif: MeJA response; GARE-motif/ TATC-box: gibberellin response; LTR: low-temperature response; MBS: MYB binding site; TCA-element: salicylic acid response; TC-rich repeats: defense and stress response; W-box: specific binding motif for WRKYs. The details of *cis*-acting elements were illustrated in supplemental Table S4

*Cis*-acting elements, significant regulators and indicators of gene function, have been comprehensively studied [40]. To investigate the diverse potential functions of *CiWRKY* genes, the Plant CARE website was used to analyze 2-kb promoter region of the *CiWRKY* genes. Each *CiWRKY* gene contained various *cis*-acting elements (Fig. 5, Table S4). A number of elements associated with stress response or hormone signaling were identified, including abscisic acid-response (ABRE), salicylic acid response (TCA), MeJA-response (TGACG/CGTCA), drought stress-response (MBS), low temperature response (LTR), and defense response (TC-rich), which are widely distributed in the promoters of the *CiWRKYs*. However, only the ABRE *cis*-acting element was present in all members of Group I, suggesting that these *WRKYs* gene may be involved in the ABA signaling pathway and have a functional role in ABA-mediated stress response. In addition, gibberellin responsive (GARE/P-box/TATC-box) elements were detected in the promoters of several *CiWRKYs*, implying that they are likely to regulate growth and development.

### Gene ontology annotation of CiWRKY proteins

**Fig. 6:**
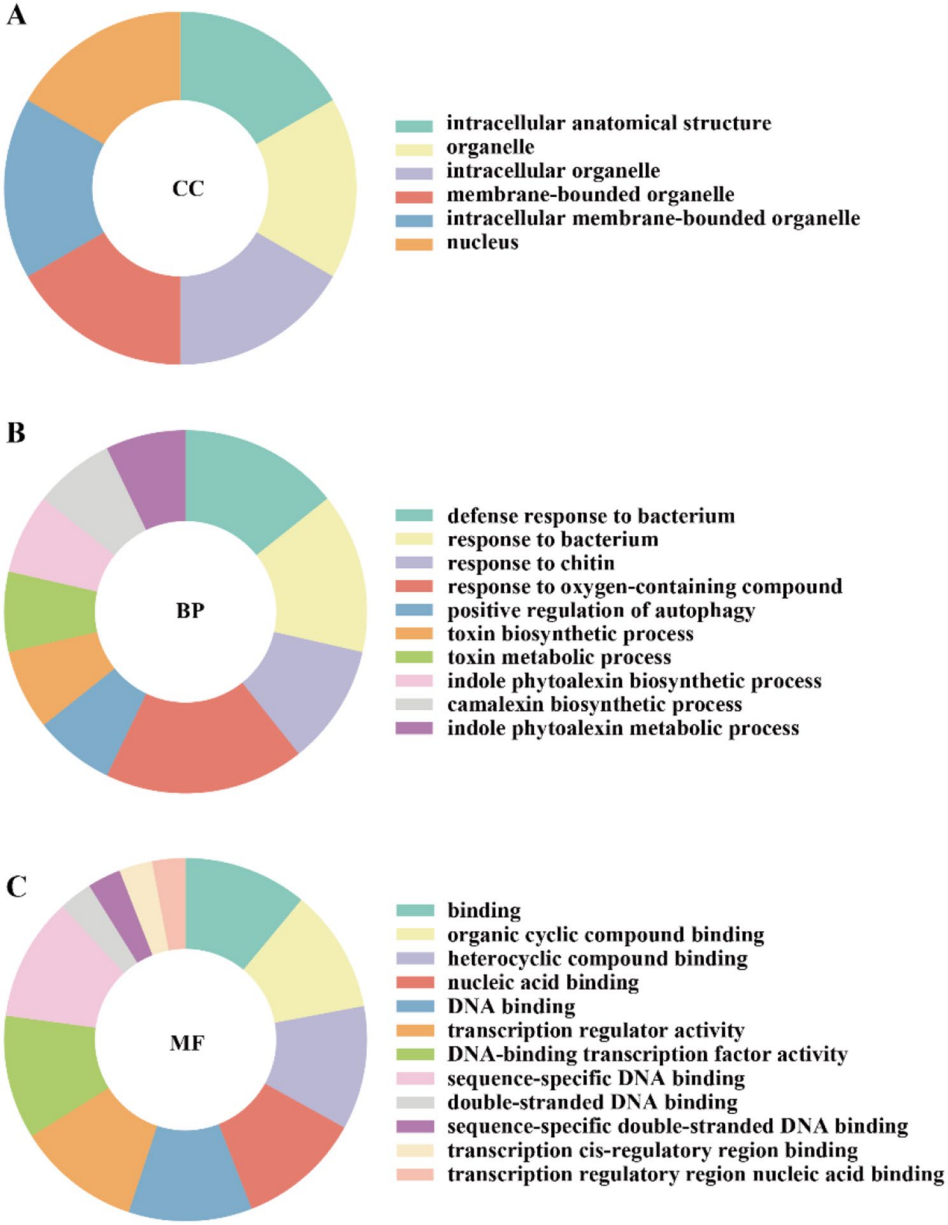
Gene ontology (GO) annotation and term enrichment of CiWRKY proteins. **(A**–**C)** Enriched GO terms of the Cellular Component (CC, **A**) Biological Process (BP, **B**) and Molecular Function (MF, **C**). GO terms with a Q-value ≤ 0.05 were considered as overrepresented. The details of GO terms were illustrated in supplemental Table S5

**Fig. 7:**
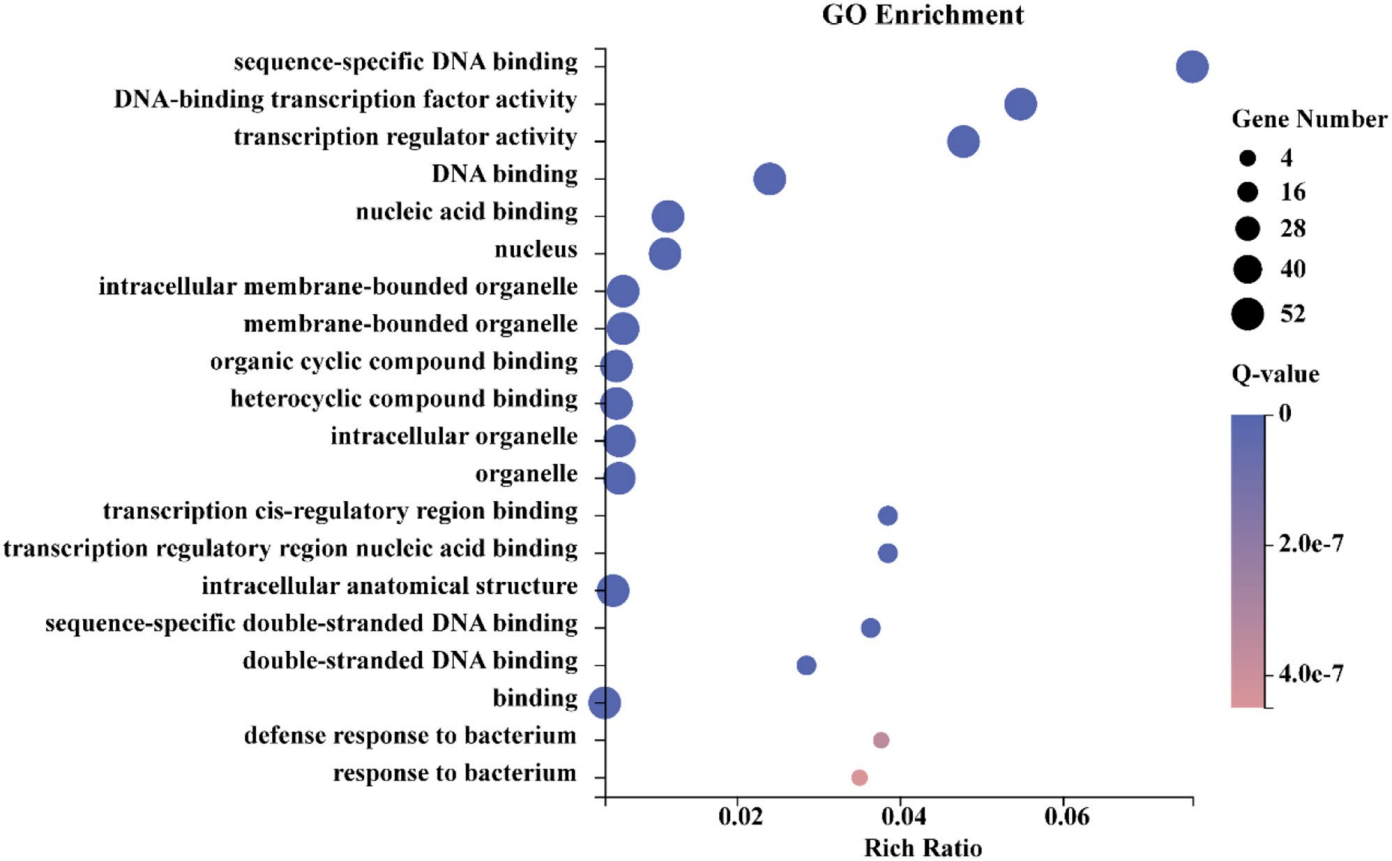
The top 20 enriched GO terms among *CiWRKY* genes. The data were analyzed using the R package and made FDR correction with Q-value ≤ 0.05. The bubble diagram was performed using the Dr. Tom Multi-omics Data Mining System. Gene number was illustrated by the size of circles. Q-value was indicated by the color shading of the bar chart

Functional annotation and predicted protein-protein interactions could gain insight into regulatory functions of the examined proteins. Gene Ontology (GO) annotations of the 52 CiWRKY proteins were scrutinized using the Dr. Tom web-based solution, an in-house customized data mining system of the BGI (Table S5). Among the protein sequences annotated in the GO database, CiWRKY proteins were categorized into three main groups, cellular component, molecular function, and biological process (Fig. 6). Six terms were greatly enriched in the cellular component category (Fig. 6A), including intracellular anatomical structure (GO:0005622), organelle (GO:0043226), intracellular organelle (GO:0043229), membrane-bounded organelle (GO:0043227), intracellular membrane-bounded organelle (GO:0043231) and nucleus (GO:0005634). In the biological process category (Fig. 6B), most CiWRKYs were found to be involved in the biological regulation (GO:0065007), response to stimulus (GO:0050896), cellular process (GO:0009987). A majority of CiWRKYs were predicted to be involved in the responses against various stimuli, indicating the crucial role of the WRKY members in modulation of stress tolerance. As for the molecular function terms the CiWRKYs were predominantly associated with binding (GO:0005488) and transcription regulator activity (GO:0140110) (Fig. 6C). The top three enriched GO terms are ‘sequence-specific DNA binding’, ‘DNA-binding transcription factor activity’ and ‘transcription regulator activity’ (Fig. 7). These results demonstrate that the CiWRKY proteins are closely involved in various biological processes, in particular the stress response.

### Protein interaction analysis of CiWRKY proteins

**Fig. 8:**
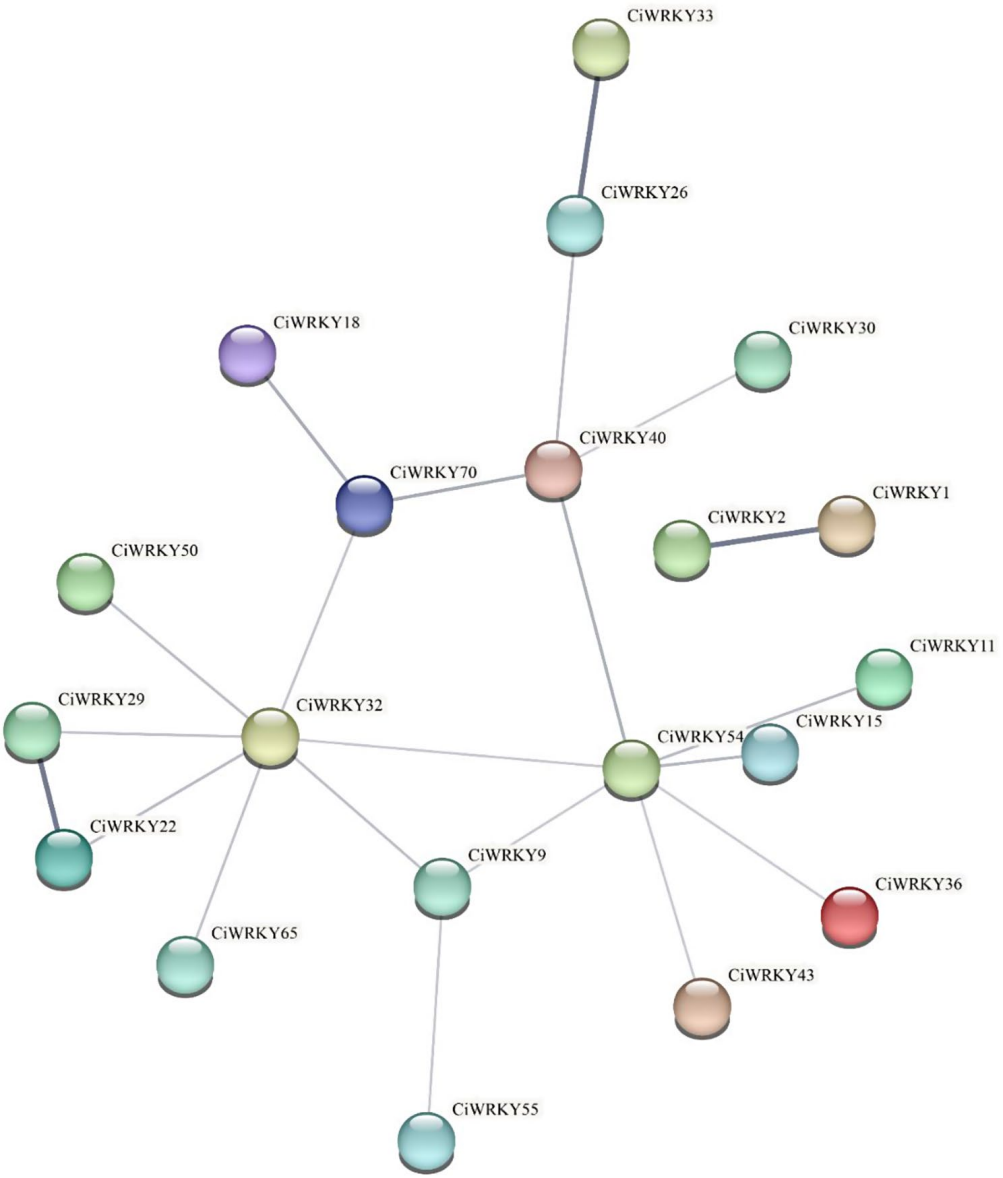
Prediction of protein-protein interactions between CiWRKYs. Protein-protein interaction of CiWRKY protein was performed by STRING online search tool. The line thickness represents the level of protein interaction

To systematically investigate the CiWRKY protein interactions, we utilized STRING 12.0 software to construct a protein interaction network (Fig. 8). As a result, interactions were identified between 20 CiWRKYs. In the network, CiWRKY33, CiWRKY26, CiWRKY29, and CiWRKY22 were associated with the plant-pathogen interactions (ath04626) and plant MAPKs (ath04016) Kyoto Encyclopedia of Genes and Genomes (KEGG) pathways. Furthermore, the interaction among CiWRKY30, CiWRKY40, CiWRKY54, CiWRKY70, and CiWRKY1 is considerably enriched with the GO terms of salicylic acid (GO:0009751), response to oxygen-containing compounds (GO:1,901,700), and chemical response (GO:0042221). Moreover, six CiWRKYs, as well as their corresponding orthologs, participated in a narrower protein interaction network.

### Expression patterns of *CiWRKY* genes in response to cold treatment

**Fig. 9:**
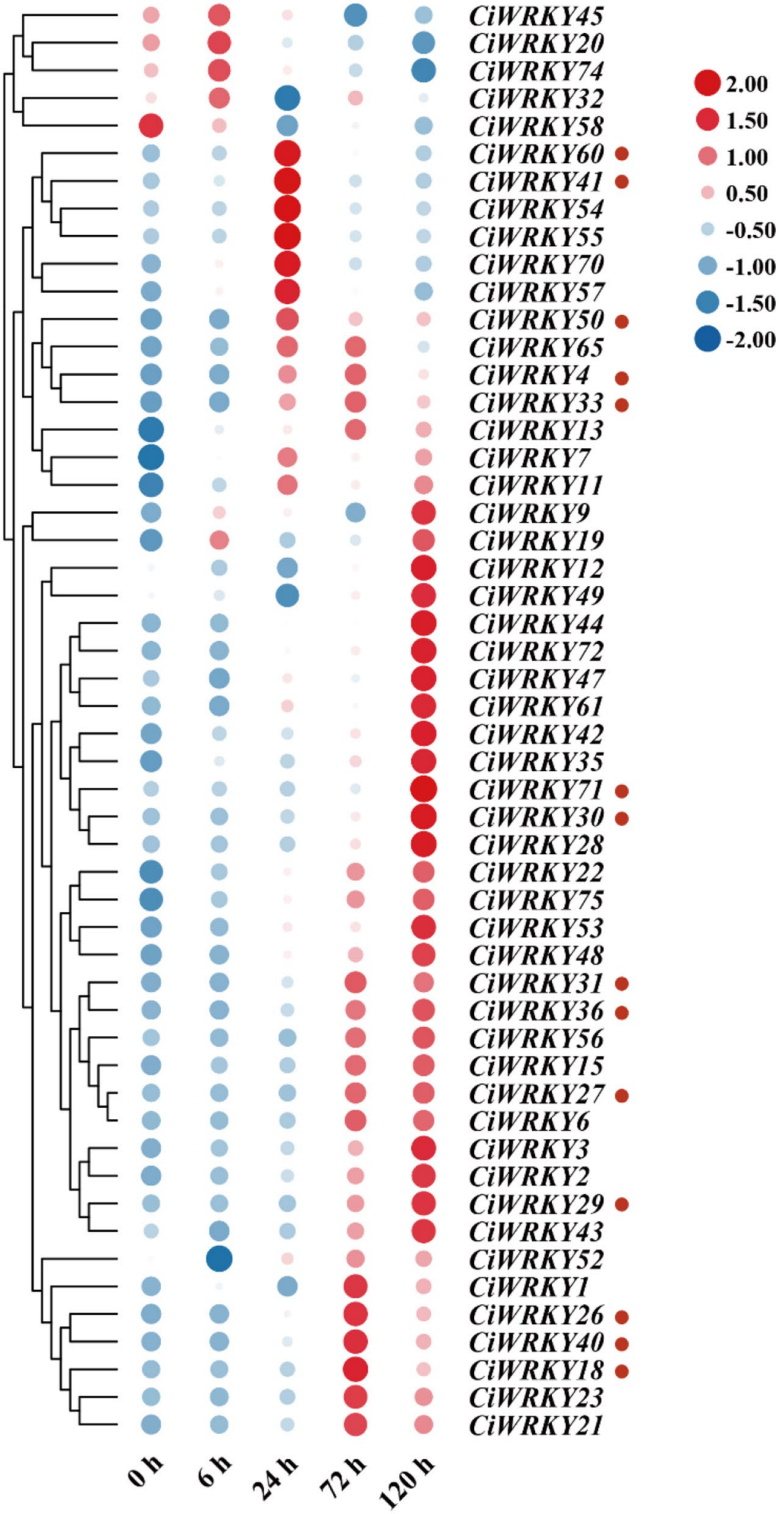
Expression profiles of *CiWRKYs* under cold treatment. Heatmap of *WRKY* gene expression in leaves subjected to cold treatment at five time points (0 h, 6 h, 24 h, 72 h, 120 h). The blue color corresponds to low expression levels, and red color corresponds to high expression levels. The heatmap was generated by TBtools. Genes that are substantially induced by cold are labeled by small red circle

**Fig. 10:**
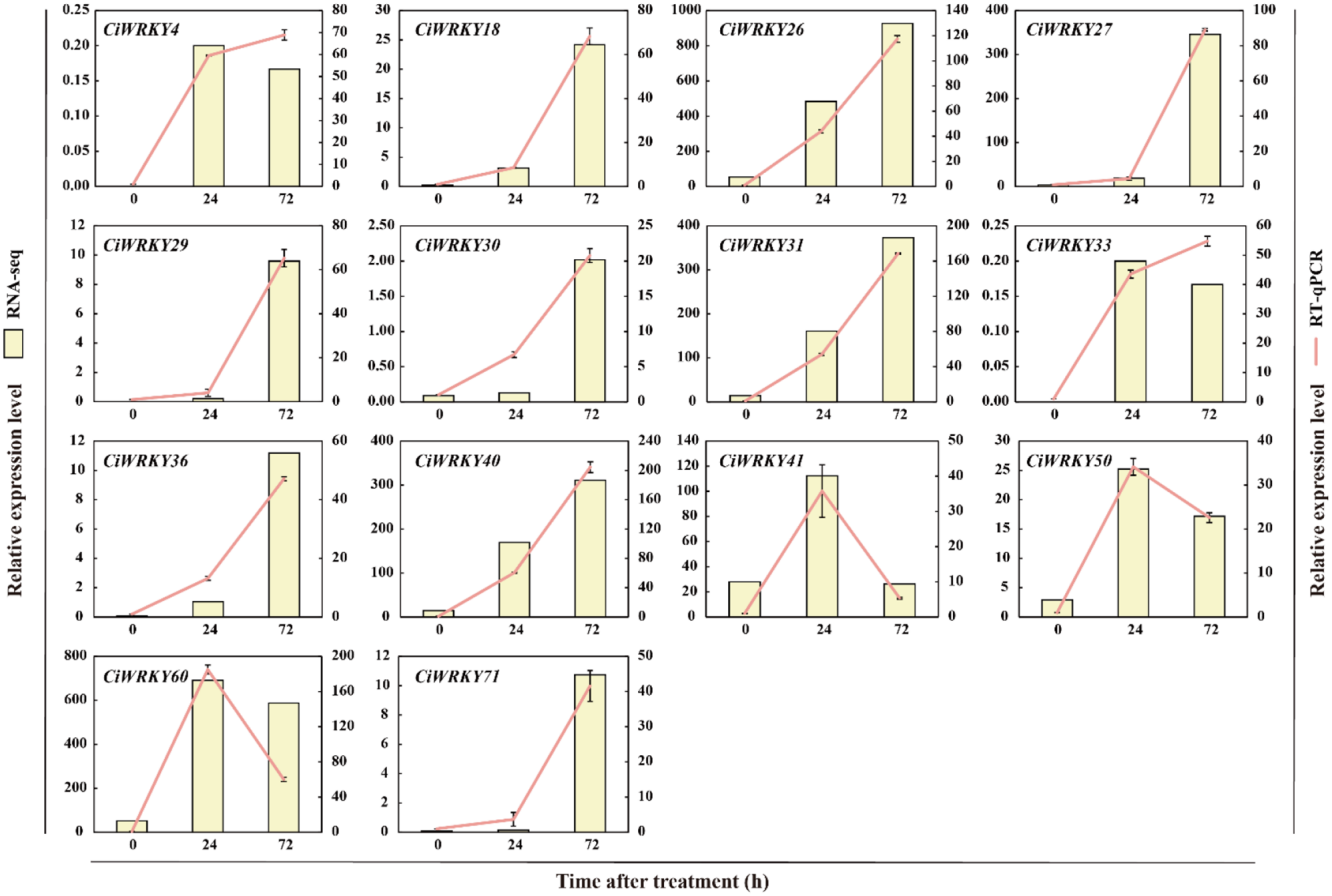
The expression patterns of 14 cold-induced *CiWRKY* genes. The relative expression of cold-induced *CiWRKY* genes was assessed by RT-qPCR (red lines) and RNA-seq (yellow columns). RT-qPCR data are means ± SD (*n* = 3)

To better understand the role of *C. ichangensis WRKYs* in cold response, leaves of three-month-old plants were sampled at five time points (0 h, 6 h, 1 d, 3 d, and 5 d) after cold treatment and subjected to RT-qPCR analysis. All of the 52 *CiWRKY* genes had detectable expression under cold stress, but displayed disparate expression patterns (Fig. 9, Table S7). Fourteen genes were substantially induced by cold stress, with stable upregulation being observed in *CiWRKY71*, *CiWRKY29*, *CiWRKY27*, and *CiWRKY30*. On the other hand, the expression levels of *CiWRKY74*, *CiWRKY58*, and *CiWRKY20* were markedly decreased upon exposure to cold, suggesting that they may have negative functions in modulation of cold tolerance. In addition, 16 *CiWRKY* genes exhibited a slight induction, while no change was detected in 19 genes under the cold stress. These results indicate that the *CiWRKY* genes may perform distinct biological functions in response to cold stress. To confirm the RT-qPCR analysis, the transcript profile of 14 cold-responsive *CiWRKY* genes in RNA-seq data [36] from Ichang papeda at four time points (0 h, 12 h, 24 h, 72 h) after cold treatment were examined (Fig. 10, Table S6), which showed that the gene expression trend was consistent between the two methods, supporting the cold-responsive nature of these genes.

### Silencing of *CiWRKY* reduced cold resistance in *C. ichangensis*

**Fig. 11:**
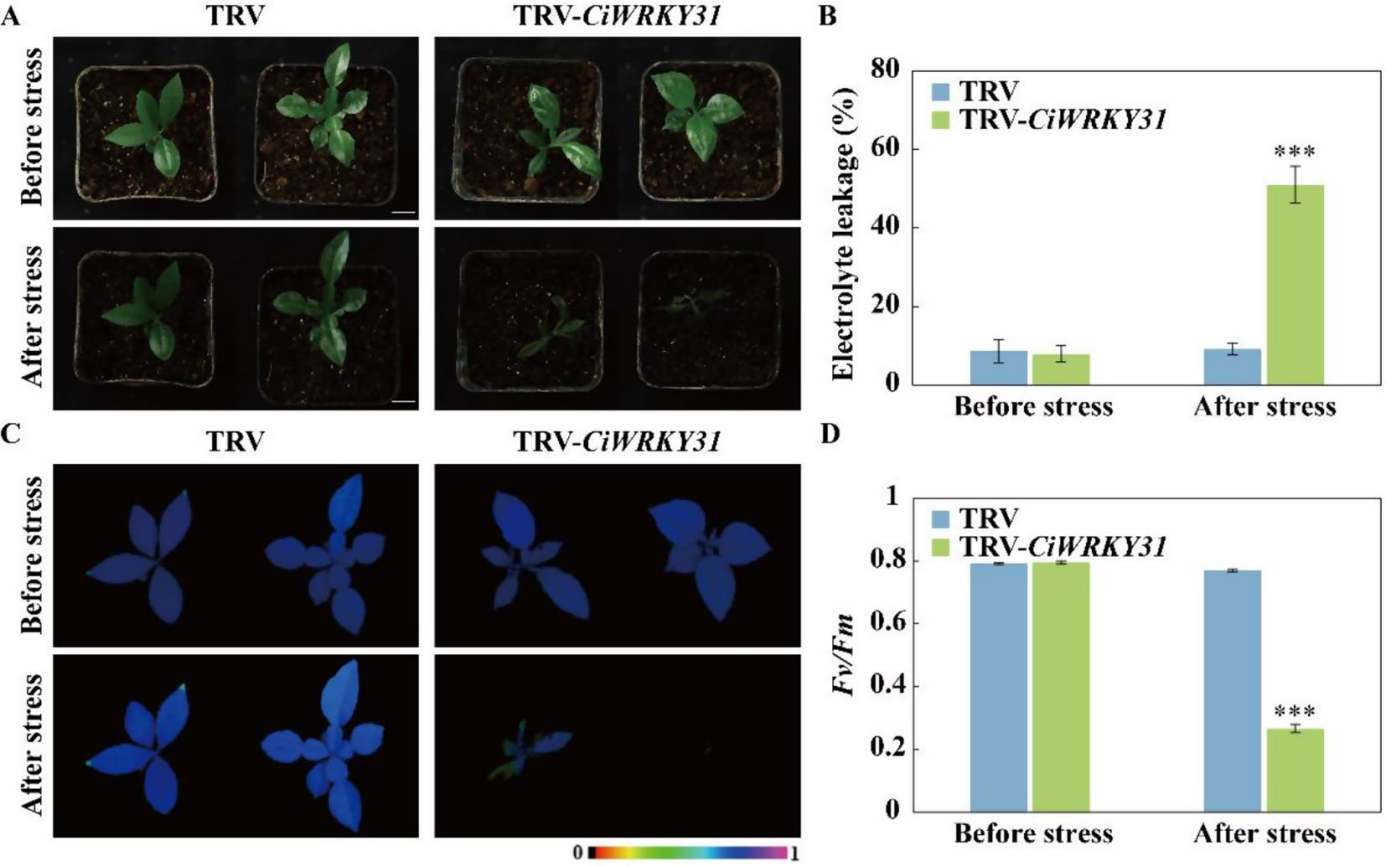
Silencing of *CiWRKY31* enhances cold sensitivity in Ichang papeda. **(A)** Phenotype of control plants (TRV), VIGS plants (TRV-*CiWRKY31*) before and after cold treatment. Scale bars = 1 cm. **(B**–**D)** Electrolyte leakage **(B)**, chlorophyll fluorescence imaging **(C)** and *Fv*/*Fm* ratios **(D)** of tested lines before and after the cold treatment. The false color scale between 0 and 1 is shown beside the imaging. Error bars indicate ± SE (*n* = 3). Asterisks indicate significant differences between the control and VIGS line under the same growth condition (*P < 0.05; **P < 0.01; ***P < 0.001)

*CiWRKY31* was found to be one of the genes that was most remarkably induced by cold stress, suggesting that it possibly plays a significant role in cold resistance. To confirm this assumption, VIGS (virus-induced gene silencing) was employed to knock down *CiWRKY31* in Ichang papeda. RT-qPCR analysis showed that the transcript levels of *CiWRKY31* were markedly down-regulated in the VIGS plants (designated as TRV-*CiWRKY31*) relative to the control plants (TRV) (Fig. S2). When subjected to freezing treatment (− 4 °C, 8 h), the VIGS plants displayed seriously visible leaf wilting, while this phenotype was not observed for the control (Fig. 11A). In agreement with the plant phenotype, EL was dramatically increase in the VIGS plants relative to the control (Fig. 11B). Meanwhile, chlorophyll fluorescence was considerably impaired, accompanied by lower *Fv*/*Fm* ratio, in the VIGS plants when compared to the control plants (Fig. 11C-D). These data indicate that knockdown of *CiWRKY31* substantially compromised the cold tolerance of Ichang papeda, indicating that *CiWRKY31* is a positive regulator of cold tolerance in Ichang papeda.

## Discussion

### *CiWRKY* genes exhibit both conserved and divergent characteristics

WRKYs, one of the largest transcription factor families in plants, are well recognized for their crucial roles in regulation of plant growth and development and response to adverse environmental stimuli [41]. Due to their valuable biological roles, *WRKY* members from several plant species have been extensively explored with the release of their whole genome sequences [42–45]. In the present study, a comprehensive analysis of the *WRKY* gene family was conducted in *C. ichangensis*, leading to identification of a total of 52 full-length *WRKY* genes. Based on phylogenetic analysis in comparison with the Arabidopsis *AtWRKYs* [19], the *C. ichangensis WRKY* members were designated *CiWRKY1* to *CiWRKY75*. Notably, the *CiWRKY* family has only 52 members, which is fewer than other angiosperms such as rice [20], soybean [21] and maize [22]. It has been reported that both *C. sinensis* and *C. ichangensis* have the same number of *WRKY* genes [46]. Furthermore, *Poncirus trifoliata*, a relative of *C. ichangensis*, also possesses a comparable number of 51 *WRKY* genes. These findings indicate that *WRKY* gene evolution in Rutaceae is probably conserved and that the *WRKY* family has not undergone significant expansion.

Physicochemical properties of the *CiWRKY* genes, including their CDS length, protein length, molecular weight, and isoelectric point, were comprehensively analyzed. Interestingly, the 52 *CiWRKY* genes exhibited substantial differences in terms of gene size. For instance, *CiWRKY58* was the largest gene, with a CDS length of 2736 bp, while *CiWRKY11* was the smallest one, whose CDS is only 240 bp. Moreover, significant variation was also observed in other properties of *CiWRKYs*, such as molecular weight and isoelectric point (Table S1). These results indicate that the *CiWRKY* family exhibits considerable diversity and is potentially involved in various cellular and developmental processes of *C. ichangensis*. To further elucidate the evolution of *CiWRKYs*, MUSCLE alignment of CiWRKY and AtWRKY domains was carried out in MEGA7 to form the neighbor-joining trees (Fig. 1). According to the phylogenetic analysis, the *CiWRKY* genes were classified into three major groups (Group I, II, III), and Group II was further group into five subfamilies (II a, II b, II c, II d, II e), which is in line with other plants [23]. The majority of the *CiWRKYs* (35) were classified into Group II (Fig. 2). The structure of conserved domains is highly similar in the members within a given group, implying that the *WRKY* family genes remained relatively stable during *C. ichangensis* evolution. Moreover, chromosomal duplication was detected, emerging throughout the *C. ichangensis* evolutionary process. Overall, 22 segmental duplication events involving 28 *WRKY* genes were identified, while there was a lack of tandem duplication events (Fig. 3). This may explain the smaller number of *CiWRKY* genes in this plant, while segmental duplications were the major driver for *WRKY* gene expansion during the evolutionary process of *C. ichangensis*.

WRKY domains, the typical structural feature of *WRKY* transcription factors, serve as a crucial point of reference in their evolutionary process [10]. It should be noted that although there is an extraordinary conservation of the WRKY domain in the *C. ichangensis WRKY* family, as 50 members possess the WRKYGQK heptapeptide. By contrast, *CiWRKY50* possesses the WRKYGKK variant, implying that there is a possibility that this gene may perform a distinct biological function (Table S1). Previous research has indicated that *AtWRKY50* promotes the production of salicylic acid and plays a role in countering defense responses induced by JA [47]. *CiWRKY50* is a cold stress responsive gene, and the unique motif structure implies that *C. ichangensis* is consistently evolving *WRKY* genes to withstand a wide range of environmental stresses.

### Gene ontology annotations and protein interactions shed light on CiWRKY functions

Gene Ontology (GO) is a standardized system for gene function classification, providing a continuously updated and comprehensive vocabulary to describe the features of genes and gene products within an organism [48]. A total of 29 GO terms were identified related to cellular component (CC), biological process (BP), and molecular function (MF) (Fig. 6). In the BP class, the top three overrepresented terms were associated with “response to stimulus”, including “defense response to bacterium”; “response to bacterium” and “response to chitin”. WRKY transcription factors play a crucial role in regulating various plant defense response, acting as transcriptional activators or repressors in a differential and graded manner [18]. Previous studies have shown their involvement in plant defense response in rice [49], pepper [50] and cotton [51], which is consistent with the GO annotation in *C. ichangensis*. This further confirms that WRKY transcription factors contribute to environmental stress tolerance. Within the CC class, six GO terms were enriched, including “nuclear”, “intracellular anatomical structure”, “intracellular organelle”, “membrane-bound organelle”, “intracellular membrane-bound organelle”, and “organelle”, suggesting that CiWRKY proteins perform highly complex functions in regulating various plant cellular processes. Within the MF category, the enriched GO terms included ‘binding’ and ‘transcription regulatory activity’, which is consist with the natures of transcription factors that have the ability to binding to specific DNA sequences. Overall, our findings suggest that *CiWRKYs* are closely involved in regulation of cellular responses to various environmental stimuli.

Protein-protein interactions can indicate the potential regulatory functions of proteins [52]. The interaction of CiWRKY proteins were identified using the STRING12 program, with the confidence parameter set at 0.4. Of these, 20 CiWRKY proteins displayed extensive interactions with other family members (Fig. 8). Five proteins, including CiWRKY9, CiWRKY32, CiWRKY40, CiWRKY54, and CiWRKY70, were positioned at the center of the network, forming a key regulatory hub that might play crucial roles in various biological processes related to the CiWRKYs. Furthermore, a significant interaction was observed between CiWRKY29 and CiWRKY22, two members in the subfamily IIe. These results implying that WRKY proteins have the potential to form homodimers or heterodimers to exert diverse functional roles. In recent years, a substantial number of WRKY-interacting proteins have been identified. For example, the interaction between WRKY33 and WRKY12 in Arabidopsis can elevate the expression of *RAP2.2*, leading to enhanced tolerance to hypoxia stress [53]. In addition, GhWRKY41 was found to facilitate a positive feedback regulation loop to improve the resistance of *V. dahliae* in cotton [54]. The extensive interactions between WRKY proteins allow for cooperative functions and promote a dynamic regulation of target genes.

### The *CiWRKY* genes may play a crucial role in cold tolerance

Recent evidence has revealed the vital functions of *WRKY* genes in response to cold stress. For instance, *VaWRKY33* was upregulated by VaERF092, which was shown to enhance cold tolerance in *Amur grape* [55]. *SmWRKY26* and *SmWRKY32* of eggplant exhibited positive regulatory functions in response to cold stress [56]. In rice, the cold-responsive *OsWRKY71* was reported to act as a transcriptional repressor that positively affected cold tolerance [57]. While the WRKY functions under cold stress have been investigated in various plant species, their involvement in cold stress resistance in *C. ichangensis* is not fully understood. In this study, the expression levels of *CiWRKY* genes were evaluated under cold stress, which revealed that the genes showed various degree of cold response. Of the genes, 14 were drastically induced by cold, 16 were slightly induced, while three genes were repressed. It is worth mentioning that 19 genes underwent negligible alterations of expression levels in the presence of cold treatment. The homologous genes of *WRKY40* and *WRKY33* in Arabidopsis have been previously shown to play significant roles in regulation of response to both biotic stresses, such as response to bacteria [58–60] or fungi [61, 62] and abiotic stresses, such as salt [37], osmotic [53], cold [63], and drought stress [64]. Some *CiWRKY* genes like *CiWRKY31* were consistently up-regulated in the presence of cold stress, implying it is possible to function in adaptation to cold treatment. The remaining highly induced genes exhibited rapid up-regulation at the onset of cold stress, peaking at either 24–72 h after the cold treatment. The diversity of expression patterns appears to be intricately linked to gene function. Due to their significance, the genes that exhibited persistent up-regulation could serve as valuable candidate to improve cold tolerance. It should be noted that three *CiWRKY* genes (*CiWRKY20*, *58*, and *74*) were significantly repressed by cold stress. *WRKY20* of wild soybean has been confirmed to positively regulate wax biosynthesis-related genes for enhancing drought tolerance [65]. Meanwhile, *OsWRKY74* has also been found to be suppressed by cold stress but strongly induced by phosphate and Fe deficiency, indicating that *WRKY74* may play a crucial role in response to nutrient starvation [66]. Additionally, *SlWRKY58* was shown to confer drought tolerance in tomato [67]. These findings corroborate the essential roles of *WRKY* genes in response to various environmental cues. To verify the expression analysis and our assumption, *CiWRKY31* was knocked down by VIGS. Silencing *CiWRKY31* significantly decreased the cold tolerance of Ichang papeda, indicating that this gene function as a positive regulator of cold stress response. This result indicates that the cold-induced *WRKYs* may play a positive role in modulation of cold tolerance, and extra work is required to elucidate the functions of other cold-responsive *WRKYs*. In addition to cold stress, *WRKY31* has been demonstrated to participate in modulation of drought tolerance [68], suggesting that *WRKY31* might have a significant biological function in combating multiple stresses.

## Conclusions

In summary, the *Citrus ichangensis WRKY* gene family was comprehensively analyzed in terms of its functional and structural properties. A total of 52 full-length *WRKY* genes were characterized and further classified into three main groups. The structure of exon-introns and the composition of motifs were similar in the same group and subgroup. Synteny and comparative phylogenetic analyses among the *WRKY* genes provided valuable insights into the evolutionary characteristics of *C. ichangensis WRKY* genes. Additionally, Gene Ontology annotation and protein-protein interaction prediction provide a foundation for further elucidate the functional properties of *CiWRKY* genes. Based on the expression patterns of *CiWRKY* genes under cold treatment we identified 14 cold-induced members, among which *CiWRKY31* was verified to be a positive regulator of cold tolerance. These findings gain valuable insights into understanding the function of *CiWRKYs* in cold stress responses, and unravel potential WRKY members that can be selected as candidates for improving cold tolerance in *Citrus*.

### Electronic supplementary material

Below is the link to the electronic supplementary material.

Supplementary Material 1

Supplementary Material 2

Supplementary Material 3

## Data Availability

The sequence information of Citrus ichangensis and Arabidopsis WRKY family genes were collected from Citrus Pan-genome to Breeding Database (http://citrus.hzau.edu.cn/index.php) and The Arabidopsis information Resource (https://www.arabidopsis.org/). All data analyzed during this study are included in this article and its additional files.
