# Supplementary material for: Genome-wide identification, expression analysis of WRKY transcription factors in Citrus ichangensis and functional validation of CiWRKY31 in response to cold stress
Source: BMC Plant Biol. 2024 Jun 28;24:617. doi: 10.1186/s12870-024-05320-0 (PMC11212357; doi:10.1186/s12870-024-05320-0)
Supplement: Supplementary file 1 — Supplementary Material 1 [file 12870_2024_5320_MOESM1_ESM.pdf]

```
                   *         20         *         40         *         60
CiWRKY1  : EVDFVNDGYRWRKYGQKLVKGNPNPRNYYRCSNS-G--CPAKKHVERASHDPKL-VITTYEGRHDHDMPPS
CiWRKY2  : EVDILDDGYRWRKYGQKVVKGNPNPRSYYKCTSA-G--CTVRKHVERASHDLKS-VITTYEGKHNHDVPAA
CiWRKY20 : EVDILDDGYRWRKYGQKVVRGNPNPRSYYKCTNA-G--CPVRKHVERASHDPKA-VITTYEGKHNHDVPTA
CiWRKY26 : DIDILDDGYRWRKYGQKVVKGNPNPRSYYKCTHP-G--CPVRKHVERASHDLRA-VITTYEGKHNHDVPAA
CiWRKY58 : EVDLLDDGYRWRKYGQKVVKGNPHPRSYYKCTNP-G--CNVRKHVERAPTDPKA-VVTTYEGKHNHDVPAA
CiWRKY32 : DVGISGDGYRWRKYGQKMVKGNPNPRNYYRCTSA-G--CPVRKHIETAVDNTSA-VIITYKGVHDHDMPVP
CiWRKY33 : DIDILDDGYRWRKYGQKVVKGNPNPRSYYKCTTT-G--CPVRKHVERASHDMRA-VITTYEGKHNHDVPAA
CiWRKY4  : DIDILDDGYRWRKYGQKVVKGNPNPRSYYKCTTT-G--CPVRKHVERASHDMRA-VITTYEGKHNHDVPAA
CiWRKY44 : DSEILSDGFRWRKYGQKVVKGNPYPRSYYRCTNP-K--CKVRKHVERASDDPRA-FITTYEGKHNHEMPLR
CiWRKY3  : EVDLLDDGYRWRKYGQKVVKGNPYPRSYYKCTTT-G--CNVRKHVERASTDPKA-VITTYEGKHNHDVPAG
CiWRKY60 : T-LIVKDGYQWRKYGQKVTRDNPSPRAYFKCSFAPS--CPVKKKVQRSAEDPSI-LVATYEGEHNHPQPTD
CiWRKY40 : TSLVVKDGYQWRKYGQKVTRDNPSPRAYFKCSFAPS--CPVKKKVQRSVEDQSV-LVATYEGEHNHPLPCQ
CiWRKY18 : S-LIVKDGHQWRKYGQKVTKDNPSPRAYFRCSMASSG-CPVKKKVQRCMEDKSF-LVATYEGEHNHDVQCS
CiWRKY31 : EAPMITDGCQWRKYGQKMAKGNPCPRAYYRCTMAVG--CPVRKQVQRCAEDRTI-LITTYEGNHNHPLPPA
CiWRKY61 : DTPTMNDGCQWRKYGQKIAKGNPCPRAYYRCTVAPS--CPVRKQVQRCAEDMSV-LITTYEGTHNHPLPVS
CiWRKY6  : EAPMINDGCQWRKYGQKMAKGNPCPRAYYRCTMAAG--CPVRKQVQRCAEDRTI-LITTYEGNHNHPLPPA
CiWRKY42 : EASMISDGCQWRKYGQKMAKGNPCPRAYYRCTMASG--CPVRKQVQRCSQDRTI-LMTTYEGNHNHPLPPA
CiWRKY47 : EAPLISDGCQWRKYGQKMAKGNPCPRAYYRCTMAVG--CPVRKQVQRCAEDMTI-LITTYEGNHNHPLPPA
CiWRKY9  : QAATINDGCQWRKYGQKIAKGNPCPRAYYRCTVAPG--CPVRKQVQRCLEDMSI-LITTYEGTHNHPLPVG
CiWRKY72 : DAPTLNDGCQWRKYGQKIAKGNPCPRAYYRCTVAPG--CPVRKQVQRCAEDMSI-LITTYEGTHSHPLPVS
CiWRKY36 : DTPTMNDGCQWRKYGQKIAKGNPCPRAYYRCTISPT--CPVRKQVQRWHEDMSI-LITTYEGTHNHPLPIS
CiWRKY12 : DVDVLDDGYKWRKYGQKIVKNSLHPRSYYRCTHN-N--CRVKKRVERLSEDCRM-VITTYEGRHNHSPCD-
CiWRKY13 : DIDVLDDGYKWRKYGQKVVKNTQHPRSYYRCTQD-N--CRVKKRVERLAEDPRM-VITTYEGRHVHSPSH-
CiWRKY19 : EIDILDDGYRWRKYGQKRDK-KIRPYYHAQWYAQ-K--AVRNTRPPR*--------------------
CiWRKY23 : EVDHLEDGYRWRKYGQKAVKDSPFPRSYYRCTSA-S--CNVKKRVERSYTDPSI-VVTTYEGQHNHPSPLL
CiWRKY28 : EIDHLEDGYRWRKYGQKAVKNSPYPRSYYRCTSQ-K--CTVKKRVERSYQDPTV-VITTYEGQHNHQCPAT
CiWRKY43 : ADDILDDGYRWRKYGQKAVKNSLYPR*---------------------------------------------
CiWRKY45 : QVDILDDGYRWRKYGQKVVKHSKFPRSYYKCTHK-G--CNVKKQVQRNTKDEEI-VVTTYEGLHTHPIGKI
CiWRKY48 : DIDHLDDGYRWRKYGQKAVKNSPHPRSYYRCTSA-G--CGVKKRVERSSEDPTI-VVTTYEGQHIHPSPIT
CiWRKY49 : -NGMADDGYKWRKYGQKSIKNSPNPRPNRKPAQQQR---SGKGPTHKLKKPKNKYLTCNKTQTRQAFPTT
CiWRKY50 : DVEILDDGFKWRKYGKKMVKNSPNPRNYYKCSVD-G--CPVKKRVERDRDDPSY-VITTYEGFHTHQSNP*
CiWRKY56 : QVDILDDGYRWRKYGQKAVKDNKFPRSYYRCTHE-G--CNVKKQVQRLTKDEGI-VVTTYEGMHNHRIEKP
CiWRKY57 : EVDHLEDGYRWRKYGQKAVKNSPFPRSYYRCTNS-K--CTVKKRVERSSEDPTI-VITTYEGQHCHHTVGF
CiWRKY71 : EVDHLEDGYRWRKYGQKAVKNSPFP----RCTTQ-K--CGVKKRVERSYEDPSI-VITTYEGQHSHPLPTS
CiWRKY75 : QVDILDDGYRWRKYGQKAVKNNKFPRSYYRCTHQ-G--CNVKKQVQRLTKDEGV-VVTTYEGMHSHPIERS
CiWRKY11 : MADIPPDDYSWRKYGQKPIKGSPHPRGYYKCSSVRG--CPARKHVERALDDPMM-LIVTYEGDHNHAF---
CiWRKY74 : VADIPPDEYTWRKYGQKPIKGSPHPRGYYKCSSVRG--CPARKHVERCPEEPSM-LIVTYEGEHNHSR---
CiWRKY21 : LADIPPDDYSWRKYGQKPIKGSPHPRGYYKCSSMRG--CPARKHVERCLEEPTM-LIVTYEGEHNHPRLES
CiWRKY15 : LSDIPPDDFSWRKYGQKPIKGSPHPRGYYKCSSVRG--CPARKHVERALDDPSM-LVVTYEGEHNHSL---
CiWRKY7  : MADIPPDDYSWRKYGQKPIKGSPHPRGYYKCSSVRG--CPARKHVERALDDPMM-LIVTYEGDHNHAF---
CiWRKY22 : -----SDVWAWRKYGQKPIKGSPYPRGYYRCSS--SKGCLARKQVERNRSDPGM-FIVTYTAEHNHPAP--
CiWRKY27 : -----ADLWAWRKYGQKPIKGSPYPRNYYRCSS--SKGCAARKQVERSNTDPNS-YIVSYTGDHTHPRP--
CiWRKY29 : -----CDKWAWRKYGQKPIKGSPYPRSYYRCSS--SKGCLARKQVERSSADPGV-FIITYGAEHNHGHP--
CiWRKY35 : EVVP-SDLWAWRKYGQKPIKGSPYPRGYYRCSS--SKGCSARKQVERSRTDPNM-LVITYTSEHNHPWP--
CiWRKY65 : ESAPPSDSWAWRKYGQKPIKGSPYPRGYYRCSS--SKGCPARKQVERSSVDPSM-LLITYSCEHNHPWP--
CiWRKY30 : ---PLDDGCSWRKYGQKDILGAKYPRGYYRCTYRNGQGCLATKQVQRSDEDPTI-FEITYRGNHTCAQASN
CiWRKY41 : ---TLDDGYCWRKYGQKDILGRNFPRGYYRCTHRHARGCLATKQVQRSDDDPSM-FEVTYRGRHTCSQNSH
CiWRKY55 : ---PPEDGYTWRKYGQKEILNSKYPRSYYRCTHQKLYKCPAKKQVQRLDDDPST-FEVVYYGDHTCHMS--
CiWRKY53 : ---PFEDGYSWRKYGQKDILGAKYPRSYYRCTYRNTQNCWATKQVQRSDEDPST-FEVTYRGTHTCFN---
CiWRKY54 : ----TDDGHAWRKYGQKVILNARFPRNYFRCTHKFDQGCQASKQVQRIQEEPPL-YRTTYYGRHTCKS---
CiWRKY70 : ----SNDGHAWRKYGQKEILNTKHPRSYFRCTHKYVQGCRATKQVQRRDDDPQM-YDTTYIGHHTCRD---
                 Dg  WRKYGqK        Pr y   c      c  k r             y g h h
```

Fig. S1 Alignment of multiple CiWRKY domain amino acid sequences.

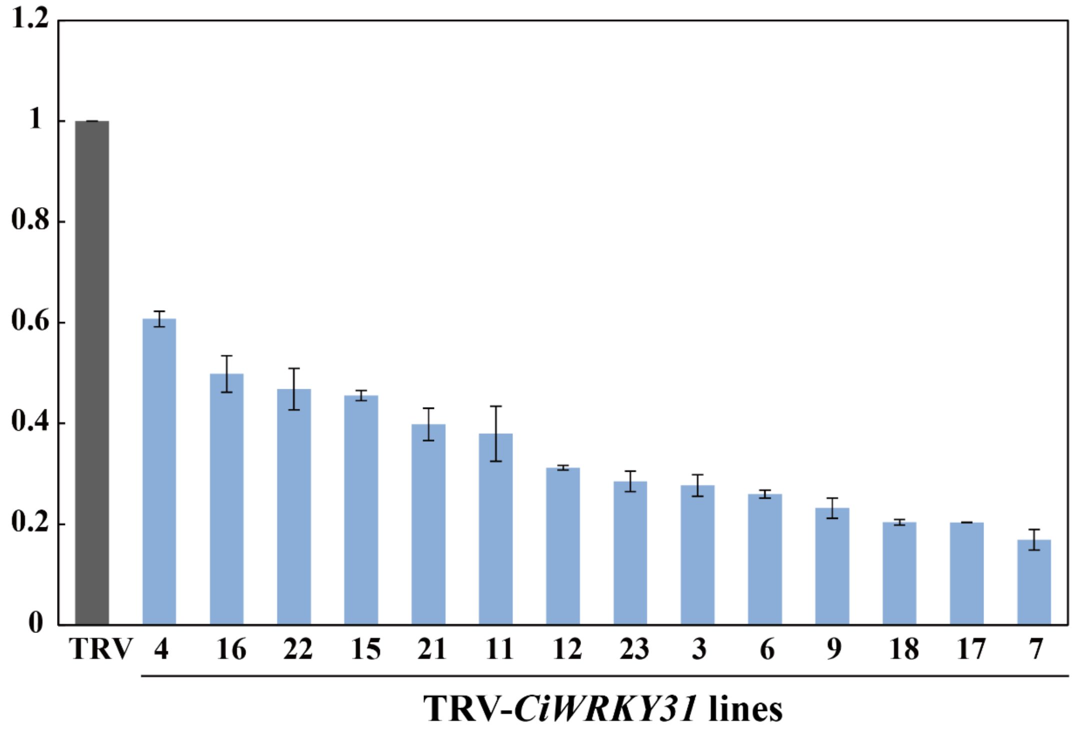

Fig. S2 Identification of TRV-*CiWRKY31* plants by RT-qPCR.
